# Are laws restricting soft drinks sales in Brazilian schools able to lower their availability?

**DOI:** 10.11606/s1518-8787.2020054001227

**Published:** 2020-04-03

**Authors:** Catarina Machado Azeredo, Maria Alvim Leite, Fernanda Rauber, Camila Zancheta Ricardo, Renata Bertazzi Levy

**Affiliations:** I Universidade Federal de Uberlândia Faculdade de Medicina UberlândiaMG Brasil Universidade Federal de Uberlândia. Faculdade de Medicina. Uberlândia, MG, Brasil; II Universidade de São Paulo Faculdade de Medicina Programa de Pós-graduação em Saúde Coletiva São PauloSP Brasil Universidade de São Paulo. Faculdade de Medicina. Programa de Pós-graduação em Saúde Coletiva. São Paulo, SP, Brasil; III Universidade de São Paulo Faculdade de Saúde Pública Departamento de Nutrição São PauloSP Brasil Universidade de São Paulo. Faculdade de Saúde Pública. Departamento de Nutrição. São Paulo, SP, Brasil; IV Universidade de São Paulo Faculdade de Medicina Programa de Pós-graduação em Saúde Coletiva São PauloSP Brasil Universidade de São Paulo. Faculdade de Medicina. Programa de Pós-graduação em Saúde Coletiva. São Paulo, SP, Brasil; V Universidade de São Paulo Faculdade de Medicina Departamento de Medicina Preventiva São PauloSP Brasil Universidade de São Paulo. Faculdade de Medicina. Departamento de Medicina Preventiva, São Paulo, SP, Brasil

**Keywords:** Adolescent, Carbonated Beverages, School Feeding, Legislation, Food

## Abstract

**OBJECTIVE:**

To describe students protected by laws and exposed to soft drinks sales and assess whether forbidding laws are associated with lower availability of these beverages.

**METHODS:**

We identified laws forbidding non-government administered cafeterias or sales of soft drinks in schools in the 27 Brazilian state capitals. Data on soft drinks sales were obtained from *Pesquisa Nacional de Saúde do Escolar 2015* (PeNSE – National Survey of School Health 2015), for a representative sample of 9th graders from public and private schools. Students were attributed with the status of their school regarding the law and sale of soft drinks. Co-variables were school status (public or private), school size, geographic regions, mother’s educational level, score of goods and services. We performed multivariate analyses using Poisson regression.

**RESULTS:**

The total of 23 laws forbidding sales of soft drinks covered 63.0% of capitals, comprising 56.9% of students. Law coverage was higher among students from more developed regions (67.6%) and in public schools (60.6%), compared with those from less developed regions (38.0%) and private schools (45.8%). Soft drinks were available for 33.9% of students. Students attending public schools in less developed regions had the lowest availability of soft drinks, regardless of law coverage (14.8%; 12.0%); while students attending private schools in these regions had a high availability, regardless of law coverage (82.1%; 73.4%). Restrictive laws were associated with lower sales of soft drinks in more developed regions, and restrictions had a greater association with the availability of soft drinks in public schools (PR = 0.25; 95%CI = 0.15-0.41), compared with private schools (PR = 0.48; 95%CI = 0.35-0.66).

**CONCLUSION:**

Laws restricting soft drinks in schools were associated with fewer sales in more developed regions. Private schools were less compliant with the law than public schools. A broadly enforced national law could reduce the availability of soft drinks in schools.

## INTRODUCTION

Sugar-sweetened beverages (SSB) are highly consumed worldwide^1 , 2^ , and soft drinks are the top source of calories from SSB among adolescents^3^ . More than one third of Brazilian adolescents reported consuming soft drinks five or more times per week^4^ , and results are similar or higher in other countries^2 , 5^ . Increased risk of obesity^6^ and death at later ages from diabetes, cardiovascular diseases and cancer have been attributted to consumption of soft drinks and other SSB^7 , 8^ .

School food environment may increase soft drinks consumption and, hence, health risks. Studies carried out in several countries found an association between the availability of unhealthy foods in schools and a higher consumption of these foods by students^9 - 12^ . This is a consequence of the easy access and the daily exposure to unhealthy foods^13^ . Therefore, laws restricting unhealthy foods and beverages in school environments might be an effective strategy to prevent and control childhood obesity and other diet-related non-communicable diseases^14 , 15^ .

Brazil does not have a national law restricting sales of foods and beverages inside schools. Nonetheless, some cities and states have approved local restrictive laws that forbid the installation of non-government administered cafeterias in schools^16^ and the sales of SSB and/or other unhealthy foods such as savory snacks and candies^17 , 18^ . Two studies that have assessed the effectiveness of these laws indicated contrasting results: while in one city the forbidden foods practically disappeared from school cafeterias^18^ ; the other study showed that around 55% of schools were still selling soft drinks^19^ .

The diversified situation of the 27 Brazilian state capitals regarding laws restricting the sales of unhealthy foods in schools enables a comparative study to assess the compliance with these laws. We thus aim to describe the Brazilian students protected by laws and exposed to sales of soft drinks and assess whether laws forbidding soft drink sales in schools are associated with lower availability of these beverages.

## METHODS

### Data source

#### Laws forbidding soft drinks sales in schools

Two trained research assistants assessed official government websites at federal, state and municipal levels to identify the existence of laws forbidding either the presence of non-government administered school cafeterias or sales of soft drinks in schools in each of the 27 Brazilian state capitals. In addition, they performed Google searches using the keywords: law, regulation, enactment, ordinance, cafeterias, canteens, school, food, soft drinks, school environment, food sales, food marketing, and the name of each state and state capital. To confirm their findings, the research assistants contacted local education authorities in each state capital where a law was found. They enquired regarding the existence, the nature, and the implementation date of the law. They have also checked whether the restriction applied to private or public schools only. A third research assistant checked the search protocol. Only laws implemented before December 31, 2014 were considered.

#### Soft drinks sales in schools

Data on soft drinks sales in schools were obtained from *Pesquisa Nacional de Saúde do Escolar* (PeNSE – National Survey of School Health 2015), carried out between April and September 2015 on a representative sample of Brazilian 9th graders from public and private schools^20^ . Each of the 27 state capitals was a stratum in the PeNSE sampling framework. The sample framework was performed in two stages: 1) schools were randomly selected in each stratum; 2) classes were randomly selected from the chosen schools. All students were invited to participate. Further details on PeNSE are available in the survey report^20^ .

We assessed 51,192 students attending 1,304 schools in the 27 Brazilian state capitals. School principals answered a questionnaire concerning school characteristics, including presence of cafeterias and soft drinks sales.

## Study variables

### Main exposure

We first classified all schools according to their status regarding the coverage of laws restricting school cafeterias or soft drinks sales. To classify schools, we considered the existence of laws in the state capital where the school was situated, and the coverage of the law (whether it was applied only to municipal public schools, only to state public schools, both municipal and state public schools, and all schools, including private schools). Then we assigned to each student the status of its school regarding the coverage of the restrictive law (e.g., if in some capital the law only covered municipal public schools, students attending state public schools in that capital were assigned as not covered by law).

### Outcome

Soft drinks sales in schools were assessed based on the principals’ report. They informed whether the school had cafeterias and soft drinks were available for sale. We classified students as attending schools selling or not soft drinks.

## Co-variables

We assessed school status (public or private); school size based on the number of students enrolled at school (up to 500, from 500 to 1000 and more than 1,000 students). We also assessed geographic regions categorized as more developed (South, Southeast and Midwest) and less developed (Northeast and North), regarding social and economic indicators; mother’s educational level (incomplete middle school, middle school, high school, college); and quartiles of a score of goods and services. The score of goods and services was created using the sum of the inverse proportion of the possessions of: mobile phone, landline phone, computer, internet, car, internal bathroom, the access to services of a housemaid^21^ .

## Statistical analysis

Our analyses were performed at student level for some reasons. First, the number of students attending each school varies, and the sample strategy of PeNSE is representative of Brazilian students, not of schools^20^ . Thus, a student level analysis is necessary to have a real estimate on the proportion of students covered by laws and exposed to sales of soft drinks. Moreover, analyses at school level could be biased, since school sample weights were not available and, therefore, each school would contribute equally to the estimates instead of proportionally to the number of students.

Descriptive analyses were performed to identify the proportion of students attending schools where soft drinks were sold, and the proportion of students covered by restrictive laws. We described these proportions according to socioeconomic and demographic characteristics (mother’s education, quartiles of score of goods, school size, school status, and geographic region).

Multiple imputation by chained equations was used to attribute numerical values to the mother’s educational level, which had 23.3% missing values (n = 11,945), as described elsewhere^10^ . The imputed data showed satisfactory statistical reproducibility according to the Monte Carlo error analysis^22^ .

We performed Poisson regression models at student level to assess the association between the exposure (student’s school status regarding the existence of laws) and the outcome (student’s school status regarding soft drinks sales). We performed Poisson models due to the high prevalence of soft drinks sales; our results therefore were described as prevalence rates^23^ . The model for total sample was adjusted for school status (private or public) and school geographic region. We also have run stratified analyses by geographic region and school status to assess whether the association between the law and the sales were different in each stratum. We found evidence of interaction; therefore, our results were presented only for population strata according the co-variables. The analyses were performed using Stata 14.1 software, and all analyses considered the sample weights and the complex sample design.

PeNSE was approved by the Research Ethics Committee (record no. 1.006.467), according to the Declaration of Helsinki, and participants gave their informed consent through a self-administered questionnaire.

## RESULTS

We found 23 laws currently forbidding either the presence of cafeterias in schools or soft drinks sales in the Brazilian state capitals. These laws covered 17 (63.0%) of the 27 state capitals, considering that in six cities (Brasília, Belo Horizonte, Curitiba, Campo Grande, Florianópolis and Rio de Janeiro), more than one restricting law was enacted.

In Belo Horizonte, Campo Grande, Florianópolis and Rio de Janeiro, the laws were from different government levels (state or city). In Curitiba and Brasília, both were state laws; the latter, however, was more detailed and comprehensive regarding which food items were forbidden. When comparing laws from Belo Horizonte and Campo Grande, the earlier enacted laws had a broader coverage of schools than the latter ones; still, both laws were considered. In this case, one was a city law covering all private and public schools, while the other was a state law, restricted to public schools.

In 12 of the 17 state capitals, available restrictions covered both public and private schools; and in the remaining five cities, only public schools were covered ( Figure ). Florianópolis and São Paulo were the first cities to enact these restrictive laws.

**Figure:**
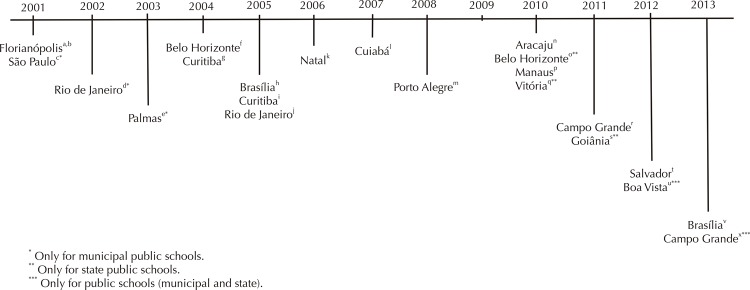
Laws Forbidding presence of canteens or soft drinks sales in schools in Brazilian state capitals. December, 2014. a. Florianópolis, SC. Lei Nº. 5.853, de 4 de junho de 2001. Dispõe sobre os critérios de concessão de serviços de lanches e bebidas nas unidades educacionais localizadas no Município de Florianópolis. Diário Oficial Município. 5 jun. 2001. b. Santa Catarina (BR). Lei Nº. 12.061, de 18 de dezembro de 2001. Dispõe sobre critérios de concessão de serviços de lanches e bebidas nas unidades educacionais, localizadas no Estado de Santa Catarina. Diário Oficial Estado, 20 dez. 2001. c. São Paulo, Secretaria Municipal da Educação. Portaria Nº 11, de 15 de fevereiro de 2001. Dispõe sobre a proibição de comércio e venda de alimentos aos alunos da Rede Municipal de Ensino, e dá outras providências. Diário Oficial do Município; São Paulo 16 de fevereiro de 2001, 46 (33), página 10. d. Rio de Janeiro. Decreto Nº 21.217, de 1 de abril de 2002. Proíbe no âmbito das unidades escolares da rede municipal de ensino adquirir, confeccionar, distribuir e consumir os produtos que menciona. Diário Oficial Município. 2 abr 2002. e. Palmas. Lei Nº. 1210, de 8 de julho de 2003. Institui e regulamenta a escolarização da alimentação escolar nas unidades escolares da rede pública municipal. Diário Oficial Município. 8 jul 2003. f. Minas Gerais. Lei Nº 15.072, de 5 de abril de 2004. Dispõe sobre a promoção da educação alimentar e nutricional nas escolas públicas e privadas do sistema estadual de ensino. Diário Legislativo. 6 abr 2004: p.26, col 1. g. Paraná. Lei Nº 14.423, de 2 de junho de 2004. Dispõe que os serviços de lanches nas unidades educacionais públicas e privadas que atendam a educação básica, localizadas no Estado, deverão obedecer a padrões de qualidade nutricional e de vida indispensáveis à saúde dos alunos. Diário Oficial Estado. 3 jun 2004; Seção 1: página 3. h. Distrito Federal. Lei Nº 3.695, de 8 de novembro de 2005. Dispõe sobre a promoção da alimentação saudável nas escolas da rede de ensino do Distrito Federal. Diário Oficial do Distrito Federal de 24 de novembro de 2005, seção 1: página 2. i. Paraná. Lei Nº 14.855, de 19 de outubro de 2005. Dispõe sobre padrões técnicos de qualidade nutricional a serem seguidos pelas lanchonetes e similares instaladas nas escolas de ensino fundamental e médio, particulares e da rede pública. Diário Oficial Estado. 20 out 2005; Seção 1: página 4. j. Rio de Janeiro (Estado). Lei Nº 4.508, de 11 de janeiro de 2005. Proíbe a comercialização, a aquisição, a confecção e a distribuição de produtos que colaborem para a obesidade infantil, em bares, cantinas e similares instalados em escolas públicas e privadas do Estado do Rio de Janeiro, na forma que menciona. Diário Oficial Estado. 12 jan 2005; Seção 1: página17 k. Natal. Lei Nº 245, de 16 de agosto de 2006 - Natal. Dispõe sobre padrões técnicos de qualidade nutricional a serem seguidos pelas lanchonetes e similares instaladas nas escolas de ensino fundamental e médio, particulares e da rede pública, e dá outras providências. Diário Oficial Município. 17 ago 2006. Seção1: página11. l. Mato Grosso. Lei Nº 8.681, de 13 de julho de 2007. Disciplina a alimentação oferecida nas unidades escolares, públicas e privadas, que atendam a educação infantil e básica do Estado de Mato Grosso. Diário Oficial Estado. 13 jul. 2007. m. Rio Grande do Sul. Lei Nº 13.027, de 16 de agosto de 2008. Dispõe sobre a comercialização de lanches e de bebidas em escolas no âmbito do Estado do Rio Grande do Sul e dá outras providências. Diário Oficial Estado. 18 ago 2008. n. Aracaju. Lei Nº 3.814, de 14 de janeiro de 2010. Dispõe sobre a alimentação oferecida nas cantinas e lanchonetes localizadas nas instituições de ensino públicas e privadas dentro da circunscrição do município de Aracaju e dá outras providências. Diário Oficial Município. 10 fev 2010. o. Minas Gerais, Secretaria Estadual de Educação. Resolução Nº 1.511, de 26 de fevereiro de 2010. Orienta a aplicação da Lei nº 18.372/2009 no âmbito das escolas do sistema estadual de ensino. Diário Oficial Estado. 27 fev 2010. Seção1: página 29. p. Manaus. Lei Nº 1.414, de 22 de janeiro de 2010. Dispõe sobre a alimentação saudável nas escolas das redes públicas e privadas de ensino na cidade de Manaus. Diário Oficial Município. 22 jan 2010. (Ano XI, Edição 2371). q. Espírito Santo. Portaria Nº 038-R, de 6 de abril de 2010. Estabelece normas para o funcionamento das cantinas escolares dos estabelecimentos da rede estadual de ensino. Diário Oficial Estado. 7 abr 2010. r. Campo Grande. Lei Nº 4.992, de 30 de setembro de 2011. Define normas para a comercialização de alimentos nas cantinas comerciais da rede pública e instituições privadas de educação básica de Campo Grande - MS e dá outras providências. Diário Oficial Município. 6 out 2011. s. Goiás (Estado), Secretaria de Estado de Educação. Portaria GAB/SEDUC Nº 3405, de 18 de maio de 2011. Resolve que fica terminantemente proibido, dentro das dependências permanentes à Secretaria de Estado de Educação, o comércio de qualquer tipo de mercadoria, seja por servidores ou por terceiros. t. Salvador. Lei Nº 8292/2012, de 16 de maio de 2012. Dispõe sobre a proibição da comercialização de lanches e bebidas de alto teor calórico que contenham gordura “trans”, nas unidades educacionais públicas e privadas. Diário Oficial do Município. 16 maio 2012. u. Roraima. Resolução Nº 001/2012/SECD/CEAE/RR. Dispõe sobre os serviços de lanches nas Unidades Educacionais Públicas que atendam a Educação básica localizadas no Estado, que deverão obedecer a padrões de qualidade alimentar e nutricional, indispensáveis à saúde dos alunos. Diário Oficial Estado Roraima. 1 ago 2012. Seção1: página 24, (ano XXII). v. Distrito Federal. Lei Nº 5146 de 19 de agosto de 2013. Estabelece diretrizes para a promoção da alimentação saudável nas escolas da rede de ensino do Distrito Federal. Diário Oficial Distrito Federal. 21 ago 2013. Seção1: página 1. x. Mato Grosso do Sul. Lei N° 4.320, de 26 de fevereiro de 2013. Proíbe a comercialização, confecção e distribuição de produtos que colaborem para acarretar riscos à saúde ou à segurança alimentar, dos consumidores, em cantinas e similares instalados em escolas públicas situadas no Estado de Mato Grosso do Sul e dá outras providências. Diário Oficial Estado. 27 fev 2013. Seção1: páginas 1-2.

Table 1 describes the students’ characteristics. Most students were from more developed regions, attended public and larger schools and had mothers with high school level.

**Table 1:** Distribution (%) of 9th grader students attending schools in the 27 state capitals in Brazil. PeNSE 2015 (n = 51,192 students).

Variables	Total of students

	% (95%CI)
Region	
Less developed	37.5 (36.0–39.0)
More developed	62.5 (61.0–64.0)
School status	
Private	27.6 (23.3–31.8)
Public	72.4 (68.2–76.7)
School size (students)	
≤ 500	14.7 (11.9–17.5)
> 500–1,000	37.1 (32.1–42.0)
> 1,000	48.2 (43.1–53.4)
Mother’s Schooling	
Incomplete middle school	23.9 (22.5–25.2)
Middle school	16.2 (15.4–17.1)
High school	34.0 (32.9–35.0)
College	25.9 (23.8–28.1)
Quartiles of SGS*	
1	27.2 (25.6–28.7)
2	27.1 (26.0–28.3)
3	32.2 (30.6–33.7)
4	13.5 (11.8–15.4)

*Score of Goods and Services.

Table 2 shows the proportion of 9^th^ year students from the 27 Brazilian state capitals that attended schools where soft drinks sales were forbidden and the proportion that attended schools where soft drinks were available for sale.

**Table 2:** Proportion of students covered by laws restricting soft drinks sales and attending schools where soft drinks were available for sale, according to sociodemographic variables. PeNSE 2015.

Variables	Students attending schools			

	Covered by law % (95%CI)	Not covered by law % (95%CI)	Selling soft drinks % (95%CI)	Not selling soft drinks % (95%CI)
Region				
Less developed	38.0 (35.6–40.3)	62.0 (59.7–64.4)	32.8 (28.4–37.2)	67.2 (62.8–71.6)
More developed	67.6 (60.7–74.5)	32.4 (25.5–39.3)	34.6 (27.0–42.3)	65.4 (57.7–73.0)
School status				
Private	45.8 (38.9–52.8)	54.2 (47.2–61.1)	68.2 (61.4–75.0)	31.8 (25.0–38.6)
Public	60.6 (54.6–66.6)	39.4 (33.4–45.4)	20.9 (15.1–26.7)	79.1 (73.3–84.9)
School size (students)				
≤ 500	53.3 (44.1–62.5)	46.7 (37.5–55.9)	38.7 (28.8–48.6)	61.3 (51.4–71.1)
> 500–1,000	62.7 (55.5–70.0)	37.2 (30.0–44.5)	26.6 (19.4–33.8)	73.4 (66.2–80.6)
> 1,000	52.7 (44.9–60.4)	47.3 (39.6–55.1)	38.1 (29.8–46.5)	61.9 (53.5–70.2)
M Schooling				
Incomplete MS	56.7 (51.4–62.0)	43.4 (38.0–48.6)	22.8 (17.7–27.9)	77.2 (72.1–82.3)
MS	57.9 (52.8–63.0)	42.2 (37.0–47.2)	27.7 (22.6–32.8)	72.3 (67.2–77.4
HS	58.7 (54.0–63.3)	42.3 (36.7–46.0)	33.9 (28.7–39.0)	66.1 (60.9–71.3)
C	52.5 (46.8–58.3)	47.5 (41.7–53.2)	48.2 (41.5–54.9)	51.8 (45.1–58.5)
Quartiles of SGS*				
1	51.1 (46.8–55.4)	48.9 (44.6–53.2)	20.7 (16.7–24.8)	79.3 (75.2–83.3)
2	60.1 (55.2–64.9)	39.9 (35.1–44.8)	31.2 (26.0–36.3)	68.8 (63.7–74.0)
3	60.3 (54.1–66.5)	39.7 (33.5–45.9)	40.1 (33.3–47.0)	59.9 (53.0–66.7)
4	51.3 (43.9–58.7)	48.7 (41.3–56.1)	51.1 (43.2–59.1)	48.9 (40.9–56.8)
**Total**	**56.5 (52.1–60.9)**	**43.5 (39.1–47.9)**	**33.9 (28.9–39.0)**	**66.1 (61.0–71.1)**

*SGS: Score of Goods and Services; MS: Middle School; HS: High School; C: College.

A little more than half of the students (56.9%) attended schools where soft drinks sales were forbidden. Coverage of laws forbidding the sales of soft drinks was higher among students from more developed regions (67.6% *versus* 38.0%) and attending public schools (60.6% *versus* 45.8%). No differences were found in law coverage regarding school size, mother’s educational level, and quartiles of score of goods and services.

A third of the students attended schools where soft drinks were available for sale. This proportion was higher among those attending private schools, with mothers at higher level of education, and in the higher quartile of goods and services.

Table 3 shows the proportion of students attending schools that sell soft drinks according to law status (covered *versus* not covered), and the association between the coverage of law and soft drinks sales stratified for the co-variables. Students from public schools in less developed regions had the lowest proportion of exposure to soft drinks sales, regardless of law (14.8% in not covered *versus* 12.0% in covered), while the private schools in these regions had a high availability, regardless of law (82.1% in not covered *versus* 73.4% in covered). In the stratified analysis, we found that the presence of restrictive laws reduced the soft drinks sales only in more developed regions. In these regions, the restrictions had a greater association with the availability of soft drinks in public schools (PR = 0.25; 95%CI = 0.15–0.41), compared with private schools (PR = 0.48; 95%CI = 0.35–0.66).

**Table 3:** Prevalence ratio (PR) of students attending schools selling soft drinks according to soft drink sales restrictions. PeNSE 2015.

Variables		Students attending schools selling soft drinks		Model

		Law status		

		Not covered	Covered	

Region	School status	%	%	PR (95%CI)
Less developed	Private	82.1	73.4	0.89 (0.72–1.11)
	Public	14.8	12.0	0.81 (0.51–1.30)
More developed	Private	85.1	41.2	0.48 (0.35–0.66)
	Public	54.8	13.6	0.25 (0.15–0.41)

## DISCUSSION

Our study provided evidence that the existence of laws in Brazilian’s state capitals was associated with a lower exposure to soft drinks sales inside schools only in more developed regions and, in these regions, we found a greater association in public schools. Around 60% of state capitals have laws forbidding sales of soft drinks in schools, covering over half of students from public and private schools.

Despite being statistically significant only in more developed regions, a negative association between the presence of restrictive laws and sales of soft drinks in schools depicts the importance of advocating for a national law in Brazil. It is noteworthy that among students attending public schools in less developed regions the exposure to soft drinks sales was low, regardless of restrictive laws, and similar to public schools covered by laws in more developed regions. This could explain the lack of association between the existence of law and the sales in public schools of less developed regions. It is of concern, however, that the presence of restrictive laws reduced only by 11% the student’s exposure to soft drinks in private schools in less developed regions (non-significant results), sustaining a high percentage of students exposed to soft drinks (73.4%).

The high proportion of students from private schools that, despite being located in cities where sales restrictions were in effect, allowed soft drinks sales in their premises (73.4% in less developed and 41.2% in more developed regions) suggests that law compliance is lower in private schools than in public schools.

Differences in law compliance have been reported for some Brazilian cities. In Belo Horizonte, more than half of the schools still sold sugary drinks (soft drinks and sugar-sweetened fruit juices) three years after the approval of the law^19^ . In Florianópolis, sales of soft drinks in schools practically stopped after five years of the law forbidding the sales of these beverages. However, sugar-sweetened fruit juices were still sold in more than 60% of the schools^17^ . This gap between the implementation of measures and the change in the supply of foods within the school environment was also found elsewhere^14 , 24^ . It seems clear that beyond the approval of laws, the government need to create mechanisms to enforce the restriction and provide healthy options for students.

A lower availability of soft drinks in schools is associated with lower consumption^25 , 26^ , and the availability of these beverages are associated with a higher consumption^10^ . Brazilian students attending schools where soft drinks were available had 13% and 23% higher regular consumption (≥ 5 times/week) in public and in private schools, respectively^10^ . In Boston, restricting the sale of sugary drinks was associated with a significant reduction, equivalent to 45 calories per day, in the consumption of these beverages among students. The success found in Boston can partly be explained by other measures taken together with the regulation, which included monitoring, nutritional education activities for parents and students, presentation of measures to school principals and negotiations with suppliers to offer healthier options in schools^26^ . These strategies contribute to the enforcement of the law and should be considered in Brazil.

The description of the food environment of public and private schools showed marked differences that are at least in part explained by the existence of the *Programa Nacional de Alimentação Escolar* (PNAE – Brazilian School Feeding Program), which provides free and nutritionally balanced meals to all students attending public schools in Brazil^27^ . Therefore, in public schools, non-government administered cafeterias sell foods that compete with meals provided by the PNAE, while in private schools’ these cafeterias are the main or only option for students to buy food. In addition, students from public schools have lower socioeconomic status than students from private schools, which may limit their possibilities to afford soft drinks or other foods from cafeterias.

Although the school environment is listed as a place for healthy eating promotion, school cafeterias in Brazil still sell ultra-processed foods and other foods of low nutritional quality. A study that used a representative sample of schools in the Federal District showed that most cafeterias did not promote a favorable environment for healthy eating, since the most commonly marketed foods were baked goods, soft drinks and sweets. In addition, the study showed that soft drinks were sold in more than 70% of the schools^28^ . Therefore, laws forbidding soft drinks sales are desirable and can reduce the consumption of soft drinks among adolescents; however, providing healthier substitutes is necessary. The promotion of water consumption, for example, could be beneficial^29^ . Taber et al.^30^ found that under strong competitive food/beverages laws, schools selling fewer unhealthy items did not provide healthy alternatives. This should be of policymakers’ concern because students may replace soft drinks for other sugar-sweetened beverages, as reported for California schools^24^ . In Brazil, this could be the case in private schools due to the presence of students with greater purchasing power.

This study has some limitations. First, we only selected state’s capitals for the analysis because PeNSE does not discriminate the other municipalities included in the sample. Nonetheless, Brazil is a highly urbanized country and almost a quarter of the population resides in state capitals^31^ . Second, PeNSE was not designed to ensure representativeness of Brazilian schools, but rather of students from these schools. Thus, our results are presented for students, not schools. However, this limitation does not interfere with the associations found. Third, some laws were not clear about what beverages were restricted therefore, we decided to exclude two dubious laws, considering that the lack of clarity would compromise the enforcement of these laws. Despite our careful search of laws, it is possible that some have not been identified. In both cases, the impact in our results would be conservative, reducing the strength of the associations.

Despite these limitations, this study still has strengths. We tested for the first time in Brazil whether the existence of laws restricting sales of soft drinks in schools were associated with the availability of such beverages, and we presented consistent results in stratified analysis. These results add evidence to the literature of middle-income countries regarding the importance of national laws aimed at reducing adolescents’ exposure to unhealthy foods. We also used a large representative sample of students and schools in the 27 state capitals in Brazil and controlled for school and individual-level factors. Finally, we performed multiple imputations to avoid selection bias due to the high proportion of missing data regarding maternal education.

We found that laws forbidding sales of soft drinks were associated with a lower presence of these beverages inside Brazilian schools in more developed regions. Although we have studied only soft drinks, it is plausible to assume the same would hold true for other unhealthy foods, since the principle is the unavailability of the item at schools. These results have important implications for public health policies in middle-income countries. From all deaths attributable to SSB consumption, 70.9% occurs in middle-income countries, while 24.1% occur in high-income and five percent in low-income countries^7^ .

Our results reinforce the need of a national law forbidding sales of unhealthy foods and beverages in schools and show the importance of law enforcement to create a supportive nutrition environment.

In conclusion, state and local policies forbidding unhealthy food sales in schools were positively associated with changes in the school food environment, only in more developed regions and with a greater reduction in availability in public schools compared with private schools. Nonetheless, the laws should be better enforced to increase school compliance, especially in private schools from both more developed and less developed regions.

Furthermore, schools provide the setting to pave the social support to promote healthy eating. Therefore, interventions to create healthy food environments in schools must be part of broader actions^32^ . Forbidding unhealthy food sales in schools is one action that should be adopted jointly to: increasing availability of healthy food choices, protecting students from in-school marketing of unhealthy foods, and promoting nutrition education. In this regard, comprehensive national policies are essential to provide adolescents with the skills and opportunities to adopt healthier eating behaviors.
